# The New Age of Adolescence and the Use of Social Networks in Youths

**DOI:** 10.5964/ejop.v15i4.2016

**Published:** 2019-12-19

**Authors:** Massimo Ammaniti, Luca Cerniglia

**Affiliations:** a Sapienza, University of Rome, Rome, Italy; b International Telematic University Uninettuno, Rome, Italy; Webster University Geneva, Geneva, Switzerland

## Abstract

Prof. Massimo Ammaniti is considered one of the most eminent Italian psychoanalysts specialized in the area of human development. In addition to carrying out his activity as a university lecturer and carrying out his profession as a psychoanalyst, Massimo Ammaniti is also the author of more than 200 scientific publications. He is a member of the International Psychoanalytical Association and he is in the Board of the World Association of Infant Mental Health. His main interests focus on parents-children interactions, adolescence, neurobiology, and the changes in the family functioning in the modern society. In this interview, he shares his thoughts about the recent transformations in the developmental stage of adolescence and reflects on the use made by youths of today’s social networks.

**Luca Cerniglia:** Professor Ammaniti, thank you very much for making time to be interviewed by me for the Europe’s Journal of Psychology. You offered a significant contribution to the field of clinical psychology and psychoanalysis, in terms of empirical research and theoretical insights and it has been an honor to have your mentorship and to work with you over the past 15 years. Your expertise in Developmental Psychopathology is wide and covers several important topics within the area of Intersubjectivity, and it is not possible to address all these subjects in a single interview. However, one of your main interests I would like to discuss with you relates to adolescence. In particular, I think that the theme of adolescents and their use of Social Networks could be interesting and stimulating for many readers.

**Massimo Ammaniti:** Yes, it is certainly one of the most important topics nowadays. You see, adolescence has greatly changed in the last years. In the past, Romans and Greeks referred to adolescence as a transition period connecting childhood with adulthood. Complex and sometimes dangerous initiation and passage rites are described in many of the texts of that period; therefore we know that the entrance into the adult world must have represented a very complex, somewhat difficult passage. Today, this perspective has (maybe luckily) changed and the passage from one developmental phase to another is less traumatic. For sure, even if today adolescents still have to pass through passage rites to become adults, these rites are established by their peers, not by their parents. The initiation rites and codes of the past are now elaborated by the group of peers: the way of dressing, wearing one’s hair and so on. This gives them a sense of sharing, of belonging, and helps teenagers face this period of transition, supporting their coping with the sense of emptiness many of them live with.

[Contrib contnr1]**Luca Cerniglia:** So, if I understand well, you are saying that the role of the family in allowing the passage from childhood to adolescence (and then to adulthood) is now played predominantly by peers?

**Massimo Ammaniti:** Yes, that is what I am saying. At the beginning of the last century, the family constituted the central place of socialization, and set the stages of the adolescent passage; and there were precise, predictable moments in this passage that sanctioned the pivotal moments of human life. These moments were much more defined than today. This helped, in some ways, the teenagers in the transition because they knew what the next step was. It also helped the parents because they knew how to behave with their offspring. There were models, and they were respected.

**Luca Cerniglia:** Do you think that changes in adolescence are related to changes in family functioning?

**Massimo Ammaniti:** Absolutely. There is no doubt that there was a change in family functioning and configuration. Just as there was a transformation in the concept of adolescence, so there was a transformation in the adolescent’s family. The family must now face a series of new tasks, and parents are no longer considered as the absolute guides; they frequently do not interfere in their children’s lives, but they rather show them their willingness to listen and orient. Of course, I am speaking with reference to what happens in industrialized countries, in Europe and particularly in Italy. The situation can be different in other countries and it is probably culturally oriented.

In the past, say until 20 or 30 years ago, adolescents vigorously opposed their parents: that was a classic Oedipal conflict between generations. Then, the scenario has changed, and adolescents now seem to need more confirmation from their parents. I think this is influenced by the fact that, in recent years, fewer children have been born into families (again, this is something more characteristic of Western and industrialized countries). These children (frequently without siblings) are at the center of the universe of their parents. A universe where generational differences are less marked than in the past. When I was a child, there were two subsystems: one including children, another one identifying parents. Now the child shares the same world of the parents. As Bauman said: the family is liquid (Bauman, 2005).

**Luca Cerniglia:** Is this a negative change, in your opinion?

[Contrib contnr2]**Massimo Ammaniti:** Unfortunately, this change has brought some negative consequences: the private space of adolescents is constantly invaded by omnipresent parents who want to become friends, confidants, accomplices to their children. The process of separation-individuation is hindered because adolescents do not have parents against whom to oppose, so the process of autonomy often translates into behaviors of rebellion or transgression. Transgressive behaviors are supported by the group of peers, which have assumed a growing weight in the life of adolescents, providing codes and meanings that are often alternative to those of the family.

Too frequently parents are afraid of being in conflict with their children and prefer to be loved rather than to maintain their own positions. This does not help adolescents: only in the diversity of points of view does a negotiation open and it is, therefore, possible to develop a solid Self.

**Luca Cerniglia:** What about Oedipus, then?

**Massimo Ammaniti:** That is exactly my point! While classical psychoanalytic theories emphasized the centrality of the Oedipal conflict that pushed adolescents against their parents, today we have adolescents-Narcissus, seeking reassurances and confirmations from their parents as well as from their peers, to narcissistically feed their fragile Self. The lexicon of adolescents has also changed; from keywords like “commitment,” “responsibility,” “work,” we now listen to other words such as “happiness,” “desires” and “personal achievement.” Moreover, the psychic world of adolescents has taken on a different configuration in which others are increasingly necessary to offer confirmations and narcissistic mirroring since the identifications with parents are lacking.

**Luca Cerniglia:** In your work, you often speak of Adultescence; can you explain what do you mean by that, and clarify if this concept is related to the above considerations?

**Massimo Ammaniti:** Well, first of all, you must consider that society has changed. The economic crisis has created great difficulties in families, career opportunities have become more difficult, and society does not give much space to adolescents. This creates an abnormal permanence of adolescents and youths in the family. Young people remain in their family house, on average, up to the age of 30 (males much more than females, who have greater autonomy and separate earlier). You can think of an adultescent as someone who is no longer an adolescent, and not yet an adult. I am not talking, of course, of physical development, but of psychological maturation. Adultescents live an uncertain, perennial present, where passive resignation is mixed with a hedonic attitude towards life. They can have children of their own, a family, and a job; however, they are stacked in an incomplete passage to adulthood. According to the Oxford Dictionary, an adultescent is “a middle-aged person whose clothes, interests and activities are typically associated with youth culture.” Adults who continue to live under the illusion of having frozen time.

**Luca Cerniglia:** I am under the impression that the digital revolution and the use of social networks would not constitute a positive factor in this already complex scenario. What do you think?

**Massimo Ammaniti:** Well, I am not sure that technology is a problem in itself. Of course, the use that adolescents do of this technology can be highly problematic.

**Luca Cerniglia:** Please, tell me more about that.

**Massimo Ammaniti:** The digital revolution has drastically changed our lives and, in particular, the life of young people: a revolution even deeper than the introduction of printing that has affected the way in which we learn, play and interact.

The first, most obvious (and possibly negative) fact associated with the technology is its overuse. The teenagers’ brains themselves become an archive of information accumulated via the Internet, tablets, smartphones, Google, Twitter, Facebook, and so on. However, Dunbar’s interesting studies (Dunbar, 2009) have shown that the human brain can effectively manage a maximum of 150 significant relationships. Beyond this limit, our mind is unable to connect with others empathically and emotionally. Instead, teenagers use to share everything on social media even with several thousands of their peers.

**Luca Cerniglia:** Yet, current research posits that adolescents feel more alone than the previous generation. What is your position on that?

**Massimo Ammaniti:** The growing loneliness of young people is a fact. Just look at a group of adolescents in a café or in a restaurant. Everyone is glued to their smartphone sending text messages. Until recently, face-to-face relationships were fundamental to create empathy, also because facial expression recognition is crucial to discern others’ intentions and feelings. In general, adolescents’ capacity for recognizing facial expressions is weaker than adults’. So, they should “practice” a lot in face-to-face interactions. Instead, social networks and online connection are usually based on text or static images (see Instagram, for instance), which do not allow the observation and recognition of the interlocutor’s facial expression. Now the new generations use emoticons to communicate their emotions, but these means are superficial and conventional. Adolescents seek to have as many contacts and likes possible but the quality of these contacts is completely lost. Just to be clear: connection is not a synonym of relationship. A relationship is founded on attachment processes; a connection falls in the realm of communication, which is very different. When we are in a relationship, we experience a contingency of time, space, and emotions. And our relationship lasts in our minds even when we are not together; we do not have to maintain a constant contact to feel a sense of intimacy. If we just connect to each other, this sense of solid closeness is very frail. That is why adolescents feel the urgency to constantly be in touch with their contacts. Some researchers say: they fear to miss out (Elhai et al., 2018). I add: they fear to miss out from their contacts’ mind.

**Luca Cerniglia:** But if the ways of being together have changed, what about the functioning of the adolescent’s brain?

**Massimo Ammaniti:** The brain (I would say “the mind”) is highly affected by this digital revolution both on a functional and structural level. The overuse and the misuse of these technologies shape young people’s brains quite deeply, influencing individual perception and experience.

**Luca Cerniglia:** How?

**Massimo Ammaniti:** This “always on” generation tends to spend many hours a day online (often even overnight) and this behavior keeps adolescents in a state of emotional, and even physiological, hyper-activation that can have very negative outcomes. These may also include a reduced capacity in attention and learning during daytime, and a general impairment in cognitive abilities. Most importantly, it has been demonstrated that the human brain needs a high-intensity functioning at and a low-intensity functioning for its healthy maturation. High intensity is usually associated with social interactions, but the brain requires also low-intensity states when the connection with others is suspended and a connection to our inner world begins. Those are the moments when we can talk to ourselves, or just wonder with our thoughts. These moments are crucial for the correct functioning and maturation of the brain, especially in childhood and adolescence. Unfortunately, social networks tend to hinder these low-intensity moments because the contact with others can be overwhelmingly constant (with dozens of notifications, text messages, voice messages, and emoticons received in a matter of minutes). Youths who are always connected experience a continuous activation that can interfere with their lives and their personal psychobiological rhythms. Hyper-activated adolescents may face difficulties in mentalizing and in emotion regulation. Thus, adolescents could face a series of problems eventually leading to social maladaptation. And the problems do not stop there.

**Luca Cerniglia:** Do elaborate please.

**Massimo Ammaniti:** Well, research has demonstrated that several neurocognitive processes are highly influenced by the environment because the structuring and the functioning of the areas of the brain underpinning these processes may be affected by context (Northoff, 2010). Accumulating literature is demonstrating that these modifications can be passed through the next generations, so in the future we could have adolescents (and then adults) less capable of empathy and of establishing and maintaining significant relationships, who would be more impulsive and more oblivious to their keen’s affective states.

**Luca Cerniglia:** Could this scenario change?

**Massimo Ammaniti:** Well, of course it could. The transformations related to digital innovations are so impacting on our life, that they have been compared with the introduction of writing in human history. Like today, innovations have been severely criticized by past intellectuals. Plato (the famous Greek philosopher) posited that writing would have had a very negative influence on the human capacity of retaining information, as people would have relied on written contents instead of strengthening their memory capability. However, history demonstrated that writing brought huge positive consequences for the development of our species and that the foreseen negative outcomes did not occur. We can speculate that something similar could happen for the use of the Internet and social networks.

**Luca Cerniglia:** Professor Ammaniti, thank you again for this conversation.

**Massimo Ammaniti:** It has been a pleasure.
